# Height, weight, and body mass index trajectories and their correlation with functional outcome assessments in boys with Duchenne muscular dystrophy

**DOI:** 10.1111/dmcn.16437

**Published:** 2025-08-31

**Authors:** Marianela Schiava, Utkarsh J. Dang, Claire Wood, Sze Choong Wong, Leanne M. Ward, Robert Muni Lofra, Anna Mayhew, William B. Martens, Stephanie Gregory, Robert C. Griggs, Michela Guglieri, the FOR‐DMD Muscle Study Group, Adnan Manzur, Amos Rachel, Andrea Gangfuss, Anne Marie Childs, Ashutosh Kumar, Barbara E. Herr, Basil T. Darras, Chris Speed, Craig Campbell, Craig M. McDonald, Ekkehard Wilichowski, Elaine McColl, Elena Pegoraro, Emma Ciafaloni, Erik K. Henricson, Federica Ricci, Gian Luca Vita, Giovanni Baranello, Giuseppe Vita, Helen Roper, Hugh J. McMillan, Iain A. Horrocks, Imelda Hughes, James F. Howard, Janbernd Kirschner, Jay J. Han, Jean K. Mah, Jeffrey M. Statland, Jennifer Wilkinson, Kate Bushby, Kathleen O’Reardon, Kevin M. Flanigan, Kimberly A. Hart, Leslie Morrison, Lorenzo Maggi, Luca Bello, Maja von der Hagen, Mary W. Brown, Mathula Thangarajh, Matthew Wicklund, Michael P. McDermott, Michelle Eagle, Monika Krzesniak‐Swinarska, Nancy L. Kuntz, Nanette Joyce, Perry B. Shieh, Peter B. Kang, Rabi Tawil, Richard J. Barohn, Richard S. Finkel, Russell J. Butterfield, Stefan Spinty, Taeun Chang, Tiziana E. Mongini, Tracey Willis, Ulrike Schara‐Schmidt, Volker Straub, W. Bryan Burnette, Wendy M. King, Zoya Alhaswani

**Affiliations:** ^1^ John Walton Muscular Dystrophy Research Centre Clinical and Translational Research Institute, Newcastle University and Newcastle Hospitals NHS Foundation Trusts Newcastle upon Tyne UK; ^2^ Department of Health Sciences Carleton University Ottawa Ontario Canada; ^3^ Great North Children's Hospital Royal Victoria Infirmary Newcastle upon Tyne UK; ^4^ Bone, Endocrine, Nutrition Research Group in Glasgow, Human Nutrition University of Glasgow Glasgow UK; ^5^ Division of Endocrinology and Metabolism Children's Hospital of Eastern Ontario Ottawa Ontario Canada; ^6^ Department of Paediatrics University of Ottawa Ottawa Ontario Canada; ^7^ Department of Neurology University of Rochester Medical Centre Rochester NY USA

## Abstract

**Aim:**

To examine the factors influencing height, weight, and body mass index (BMI) z‐scores, and the relationship between them and motor performance, in boys with Duchenne muscular dystrophy (DMD).

**Method:**

This was a randomized, double‐blind, parallel group trial involving 32 study sites across five countries. Height, weight, BMI z‐scores, and clinical outcome assessments (COAs)—rise from supine velocity, 10‐m walk/run velocity, NorthStar Ambulatory Assessment, and 6‐minute walk test—were analysed in 4‐year‐old to 7‐year‐old boys with DMD randomized to 0.75 mg/kg/day prednisone, 0.75 mg/kg/day intermittent prednisone, or 0.90 mg/kg/day deflazacort in the FOR‐DMD study. Trajectories were modelled using a linear mixed‐effects model and correlations were explored through Spearman's partial correlations.

**Results:**

In 194 boys with DMD, higher height at glucocorticoid initiation was associated with slower growth (*p <* 0.001) and older age was associated with increased weight gain (*p* = 0.001). Glucocorticoid type and regimen influenced height and weight trajectories but not BMI. Changes in height and weight z‐scores were negatively correlated with COAs (*p <* 0.05 in all cases). Correlations were weak 3 years after glucocorticoid initiation and moderate after 5 years (closer to the age of loss of ambulation).

**Interpretation:**

Changes in anthropometric measures after glucocorticoid initiation are associated with COA performance and larger correlations closer to the age of loss of ambulation. This emphasizes the need for weight management strategies and discussions that support treatment.

## Graphical abstract

The analysis of height, weight, and BMI z‐score trajectories in boys with DMD from the FOR‐DMD study showed that higher baseline height was associated with slower subsequent growth, and older age with greater weight gain after glucocorticoid initiation. Changes in height and weight z‐scores were negatively correlated with motor performance outcomes. These findings support clinical discussions and highlight the importance of proactive weight management in optimizing functional outcomes in DMD.

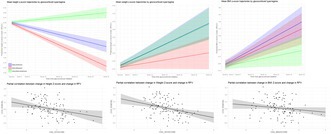

**This original article is commented on by Brogna and Mercuri on pages** 310–311 **of this issue.**

Plain language summary: https://onlinelibrary.wiley.com/doi/10.1111/dmcn.70012

**Abbreviations**
COA: clinical outcome assessment
DMD: Duchenne muscular dystrophy
NSAA: NorthStar Ambulatory Assessment
RFV: rise from supine velocity
10MWRV: 10‐m walk/run velocity

**What this paper adds**

- Height at glucocorticoid initiation is a better predictor of growth trajectory than age.
- Older age is associated with greater weight gain after glucocorticoid initiation.
- Growth metrics and clinical outcome measures are correlated in Duchenne muscular dystrophy (DMD).
- Height and weight changes after glucocorticoid treatment affect clinical outcome assessments.
- Weight control is key to preserve function as DMD progresses.

Duchenne muscular dystrophy (DMD) is a genetic disorder caused by variants in the *DMD* gene, resulting in dystrophin deficiency.^1^ Glucocorticoids are part of the standard of care^1^ and are recommended to be started early to optimize long‐term motor, cardiac, and respiratory function.^1^, ^2^, ^3^, ^4^

Chronic glucocorticoid use in DMD causes growth failure and short stature, affecting self‐image.^5^ While newborn infants with DMD have average birth length,^6^ growth slows by ages 3 to 4 years^6^ and remains below typically developing peers.^7^, ^8^ Glucocorticoid‐induced lack of pubertal growth spurts^9^ and prolonged use further exacerbate height stunting.^10^

The effects of glucocorticoids on growth and anthropometry depend on type and regimen. Daily dosing affects growth more than intermittent dosing (10 days on, 10 days off). Deflazacort affects growth more than prednisone but is linked to lighter weight.^3^ Higher cumulative doses and longer use correlate with shorter stature (both drugs) and greater weight (prednisone).^11^

Emerging data link specific *DMD* gene variants, particularly those affecting the Dp71 isoform, to impaired growth.^12^ Dp71, a ubiquitous isoform, has roles in cell adhesion, cell division, water homeostasis, and nuclear architecture, potentially affecting growth plate cells and contributing to growth failure.^12^

The association of anthropometric measures (height, weight, and body mass index [BMI] z‐scores) and motor performance in ambulant boys with DMD after glucocorticoid initiation is unclear. Taller and overweight boys may experience worse motor performance and earlier loss of ambulation.^11^, ^13^, ^14^ Analysis of the Cooperative International Neuromuscular Research Group^13^ and UK NorthStar^14^ cohorts showed that shorter stature correlates with longer ambulation. A mathematical model suggests that shorter stature diminishes the load per muscle fibre, potentially mitigating disease severity.^15^ Those studies explored the effect of height on loss of ambulation; however, it is uncertain if changes in anthropometrics over time or final measures correlate with clinical outcome assessments (COAs) and when their influence on motor performance is significant.

This study aimed to: (1) describe height, weight, and BMI z‐score trajectories after glucocorticoid initiation in a large cohort of young boys with DMD; (2) investigate the association of different factors, such as dystrophin isoforms, age at glucocorticoid initiation, cumulative dose of glucocorticoid, and baseline anthropometric measures with growth in young boys with DMD treated with glucocorticoids; and (3) investigate the correlation between changes in height, weight, and BMI z‐scores, and changes in COAs, in the 3‐year and 5‐year period after glucocorticoid initiation, and assess the correlation between absolute height, weight, and BMI z‐score values and COAs after 3 years and 5 years of glucocorticoid treatment.

## METHOD

### The FOR‐DMD study

The FOR‐DMD study was a randomized, double‐blind, parallel group trial involving 32 study sites across five countries (ranked here from most to least enrolled): the USA, the UK, Italy, Germany, and Canada. The study protocol was published elsewhere.^3^ Briefly, eligible participants were glucocorticoid‐naive boys with clinical signs of DMD, a confirmed pathogenic variant in the *DMD* gene, aged between 4 years and up to 7 years and 11 months at the time of screening, and able to rise independently from the floor.^3^ Eligible participants were randomized with equal allocation to receive: (1) daily 0.75 mg/kg/day prednisone; (2) intermittent 0.75 mg/kg prednisone (10 days on, 10 days off; hereafter intermittent prednisone); or (3) daily 0.90 mg/kg/day deflazacort. After baseline, follow‐up visits were scheduled at 3 and 6 months and every 6 months thereafter.

A total of 196 boys were recruited with a planned study duration of 36 months. For those who were recruited early, they could be followed for up to 2 years longer.

Height, weight, and BMI z‐scores were available for 194 prepubertal boys (1284 total observations over 3 years). Two boys were excluded because they were screened but not dosed and 128 observations belonging to 50 boys were excluded because the percentage of target dose of glucocorticoids dropped 25% or more between baseline and year 3.^2^

### Study design and procedure

For the present study, data for up to 5 years of follow‐up were analysed. The variables of interest were age at glucocorticoid initiation (in years) and COAs, including rise from supine velocity (RFV) (rise per second), 10‐m walk/run velocity (10MWRV) (10 m per second), NorthStar Ambulatory Assessment (NSAA) total score, and 6‐minute walk test (m). If a baseline value was missing, then the screening value was substituted for the baseline value. Dystrophin isoforms were grouped based on the effects of the *DMD* variant on dystrophin isoform expression (ref. sequence NM_004006.3, genotype) according to the rationale outlined by Stimpson et al.:^16^ group 1 (Dp427 absent, Dp140/Dp71 present); group 2 (Dp427/Dp140 absent, Dp71 present); and group 3 (Dp427/Dp140/Dp71 absent). The glucocorticoid regimen and type followed the FOR‐DMD protocol as explained earlier.^3^ The glucocorticoid cumulative dose was the daily dose in mg/kg/day multiplied by the number of treatment days. This calculation was done at each visit, and the results were added together to obtain the cumulative dose. The total cumulative dose over the 36‐month follow‐up period was then divided by 100 to scale the values. To facilitate comparisons, deflazacort doses were converted to prednisone equivalents using the ratio of 0.75 mg/kg/day prednisone to 0.90 mg/kg/day deflazacort.^17^, ^18^ The 2018 standard of care recommends a minimum of 25% reduction in glucocorticoid dose^2^ if required because of side effects. To ensure the analysis of boys who maintained a similar glucocorticoid percentage of the target dose throughout the study period, observations after a dose reduction of 25% or more were excluded from the analysis. Anthropometric measurements included height, weight, and BMI z‐scores. Standing height was measured using a wall‐mounted stadiometer, with boys standing barefoot, heels against the wall, and arms at their sides. The same person took the measurement for consistency. Boys with Achilles tendon contractures were measured using both standing height and ulna length. In non‐ambulant boys, ulna length was used for height estimation. Weight was recorded in kg using a calibrated scale, with non‐ambulant boys weighed on a digital sitting scale, both without orthoses and wearing light clothing. BMI was calculated as weight (kg)/height^2^ (m^2^) and expressed in absolute values. All anthropometric data were converted into z‐scores using the Centers for Disease Control and Prevention growth charts for the USA.^19^

### Standard protocol approvals, registration, and patient consent

The FOR‐DMD study was registered at ClinicalTrials.gov (registration no. NCT01603407); the required regulatory approvals were obtained at each participating country and site.^3^ Consent from parents or legal guardians and assent from the boys (when appropriate) were obtained at the screening visit before performing any study assessments according to good clinical practice. This work is the authors' own, and no copyright has been breached.

### Statistical analysis

To investigate the effect of dystrophin isoform on height z‐score trajectories up to 3 years of follow‐up, a linear mixed‐effects model was fitted that included the following factors as fixed effects: the intercept; the dystrophin isoforms; month of follow‐up as the time metric; and the interaction between dystrophin isoforms and month. The model also included random effects for intercept and month. Estimated marginal means of height z‐score at baseline and in the 3‐year period were computed using the emmeans package v1.8.8 in R to assess the adjusted means for each level of dystrophin isoform. The influence of dystrophin isoforms on weight or BMI was not analysed because of lack of supporting literature.

To investigate the height, weight, and BMI z‐score trajectories up to 3 years of follow‐up according to glucocorticoid regime and type, a linear mixed‐effects model was fitted for each anthropometric measure that included the following exploratory variables as fixed effects: the height, weight, or BMI z‐score at the time of glucocorticoid initiation (thereafter, baseline z‐score); age at glucocorticoid initiation; glucocorticoid cumulative dose; glucocorticoid regime and type; month of follow‐up as the time metric; and the interactions between factor and month. Boys on daily prednisone served as the reference category for this model. The model also included random effects for intercept and month. This model did not include the dystrophin isoform because of non‐significant findings in the aforementioned analysis.

An additional model was fitted that included the baseline z‐score, the cumulative glucocorticoid dose, and a three‐way interaction between age at glucocorticoid initiation categorized as below or above 6 years old, glucocorticoid regimen and type, and month as fixed factors. Age at glucocorticoid initiation was categorized as below or above 6 years old because a previous subanalysis of the FOR‐DMD study identified that this age cut‐off significantly influenced the COA trajectories.^7^ This analysis aimed to examine the 3‐year height, weight, and BMI z‐score trajectories across all six possible combinations between age at glucocorticoid initiation and glucocorticoid regime and type. For this analysis, the category that had the least impact on the specific outcome was used as the reference category. For the height and BMI z‐score model, boys below the age of 6 years on intermittent prednisone served as the reference category. For the weight z‐score model, boys below the age of 6 years on daily deflazacort served as the reference category. The mean height, weight, and BMI z‐scores in the 3‐year period were calculated for each of the six groups, using Bonferroni correction for multiple comparisons. This model did not include the dystrophin isoform because it was not significant in the aforementioned analysis.

Mean height, weight, and BMI z‐scores over the 3‐year period were compared across different glucocorticoid regimens and types using the estimated marginal means. Adjustments were made for the baseline z‐score, age at glucocorticoid initiation, and cumulative glucocorticoid dose. Bonferroni corrections were applied for multiple comparisons.

To examine the correlation between height, weight, or BMI z‐scores and COAs, we conducted Spearman's partial correlations. The analysis included two approaches: (1) determine whether the change from baseline in height, weight, or BMI z‐score correlated with the change in COA in a 3‐year and 5‐year period; or (2) determine whether height, weight, or BMI z‐scores at year 3 or 5 correlated with the COA values in those years. The Spearman's partial correlation analysis was adjusted for age at glucocorticoid initiation, glucocorticoid cumulative dose, baseline COA performance, and glucocorticoid regime and type. Loss of ability to rise from the floor was defined as not being able to complete the task, with a velocity of zero allocated to those unable to perform it.^1^ Loss of ambulation was defined as scoring 0 (‘unable to achieve independently’) on item 2 (‘walk’) of the NSAA.^3^

A level of significance of 0.05 was used for hypothesis testing. All statistical analyses were performed using R (R Foundation for Statistical Computing, Vienna, Austria). The following R packages were used: lme4 v1.1–35.3, emmeans v1.8.8, and correlation v0.8.4.^20^, ^21^, ^22^, ^23^, ^24^

## RESULTS

The baseline characteristics in our cohort are summarized in Table 1.^3^ No boys received growth hormone during the study period; in no case a past medical history of a condition that could adversely affect growth was reported by the clinicians at baseline. The study protocol mandated annual assessment of vitamin D levels and vitamin D supplementation as per national guidelines. Vitamin D levels through the study are available in Appendix S1.

**TABLE 1:** Baseline characteristics of the FOR‐DMD boys included in this study.

Variable	3‐year follow‐up, *n* = 194	5‐year follow‐up, *n* = 32
Country, *n* (%)		
USA	82 (42.3)	9 (28.1)
UK	58 (30.0)	18 (56.2)
Italy	22 (11.3)	2 (6.2)
Germany	20 (10.2)	3 (9.4)
Canada	12 (6.2)	0 (0)
Age at glucocorticoid initiation (years), mean (SD) (median, minimum–maximum)	5.8 (1.0) (5.7, 4.1–8.1)	5.5 (1.0) (5.6, 4.1–7.8)
Baseline z‐scores, mean (SD) (median, minimum–maximum)		
Height	−0.9 (1.0) (−0.9, −3.1 to 2.2)	−0.8 (0.9) (0.7, −2.6 to 0.7)
Weight	−0.3 (1.0) (−0.4, −3.0 to 2.4)	−0.3 (0.9) (−0.3, −2.3 to 1.5)
BMI	0.5 (1.0) (0.6, −2.8 to 3.0)	0.4 (1.0) (0.6, −2.7 to 2.0)
Baseline clinical outcome assessment performance, mean (SD) (median, minimum–maximum)		
6MWT (m)	336.7 (61.0) (344.0, 102.0–481.0)	328.0 (79.5) (340.0, 125.0–469.9)
NSAA (total score)	21.4 (5.3) (21.0, 6.0–34.0)	21.0 (5.2) (20.0, 12.0–31.0)
RFV (rise per second)	0.18 (0.08) (0.17, 0–0.47)	0.19 (0.08) (0.17, 0.05–0.40)
10MWRV (10 m per second)	0.17 (0.04) (0.16, 0–0.26)	0.16 (0.04) (0.16, 0.06–0.25)
Dystrophin isoform deficiency, *n* (%)		
Group 1 (Dp427 absent, Dp140/Dp71 present)	83 (42.8)	15 (47.0)
Group 2 (Dp427/Dp140 absent, Dp71 present)	46 (23.7)	8 (25.0)
Group 3 (Dp427/Dp140/Dp71 absent)	3 (1.5)	1 (3.1)
Uncertain effect on dystrophin isoform expression	62 (32.0)	8 (25.0)
Glucocorticoid regime or type, *n* (%)		
Daily prednisone	64 (33.0)	14 (43.8)
Daily deflazacort	64 (33.0)	9 (28.1)
Intermittent prednisone	66 (34.0)	9 (28.1)
Glucocorticoid progressive cumulative dose (mg), mean (SD) (median, minimum–maximum)		
Daily prednisone	635.0 (80.0) (631.6, 448.0–810.0)	4183.4 (449.8) (4267.2, 3563.6–4851.6)
Daily deflazacort^a^	650.0 (83.5) (631.5, 507.0–831.0)	4872.8 (1007.5) (5252.5, 3443.9–5844.1)
Intermittent prednisone	311.0 (36.0) (302.0, 266.0–397.0)	1897.1 (205.0) (1831.4, 1700.4–2227.8)

^a^ The deflazacort dose was converted to prednisone to facilitate comparisons. Equivalence: 0.9 mg/kg/day deflazacort = 0.75 mg/kg/day prednisone. Age reported in decimal years for consistency with other papers using the FOR‐DMD data.^4^, ^7^

Abbreviations: 6MWT, 6‐minute walk test (m); 10MWRV, 10‐m walk/run velocity; BMI, body mass index; NSAA, NorthStar Ambulatory Assessment; RFV, rise from supine velocity.

### Factors influencing growth trajectories after 3 years of glucocorticoid in boys with DMD

**FIGURE 1:**
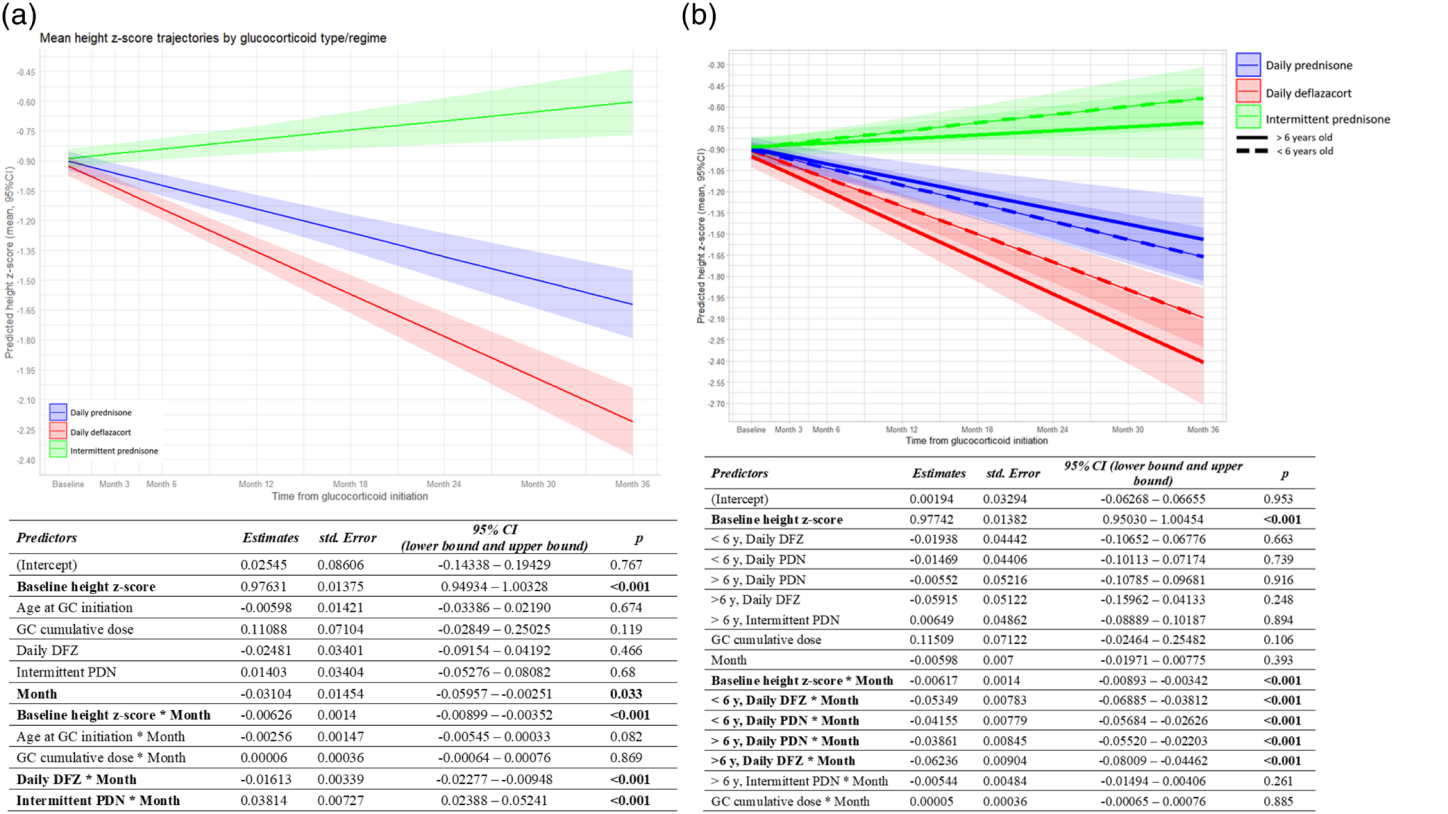
Height z‐score trajectories. (a) Line graphs from the linear mixed model analyses showing height z‐score trajectories over the 3‐year period of the FOR‐DMD study, categorized according to glucocorticoid regimen and type. The shading represents the confidence interval around the predicted trajectory, as estimated using the linear mixed‐effects model. The reference category consisted of boys on a daily prednisone regimen. (b) Line graphs from the linear mixed‐effects model analyses showing height z‐score trajectories over the 3‐year period of the FOR‐DMD study, categorized according to glucocorticoid regimen and type, and age at glucocorticoid initiation, either below or above 6 years old. The reference category consisted of boys below the age of 6 years on intermittent prednisone. Despite older boys at the time of glucocorticoid initiation grew less in the 3‐year period compared to younger boys, the effect of age at glucocorticoid initiation on the height trajectory was not statistically significant, as shown in (a). This was further confirmed in the linear mixed‐effects model analysis (b). There were no significant differences in the mean height z‐scores over the 3‐year period between boys below or above 6 years old in the same glucocorticoid group (mean [standard error] height z‐score change in the 3‐year period: less than 6 years daily PDN −1.248 [0.058] vs more than 6 years daily PDN −1.198 [0.076], mean difference 0.050 [0.084], *p* = 1; less than 6 years daily DFZ −1.419 [0.058] vs more than 6 years daily DFZ −1.582 [0.079], mean difference − 0.163 [0.084], *p* = 0.057; less than 6 years intermittent PDN −0.655 [0.081] vs more than 6 years intermittent PDN −0.724 [0.083], mean difference − 0.069 [0.080], *p* = 1). The variables in bold indicate those that achieved statistical significance in the model. Abbreviations: CI, confidence interval; DFZ, deflazacort; GC, glucocorticoid; PDN, prednisone/prednisolone.

**FIGURE 2:**
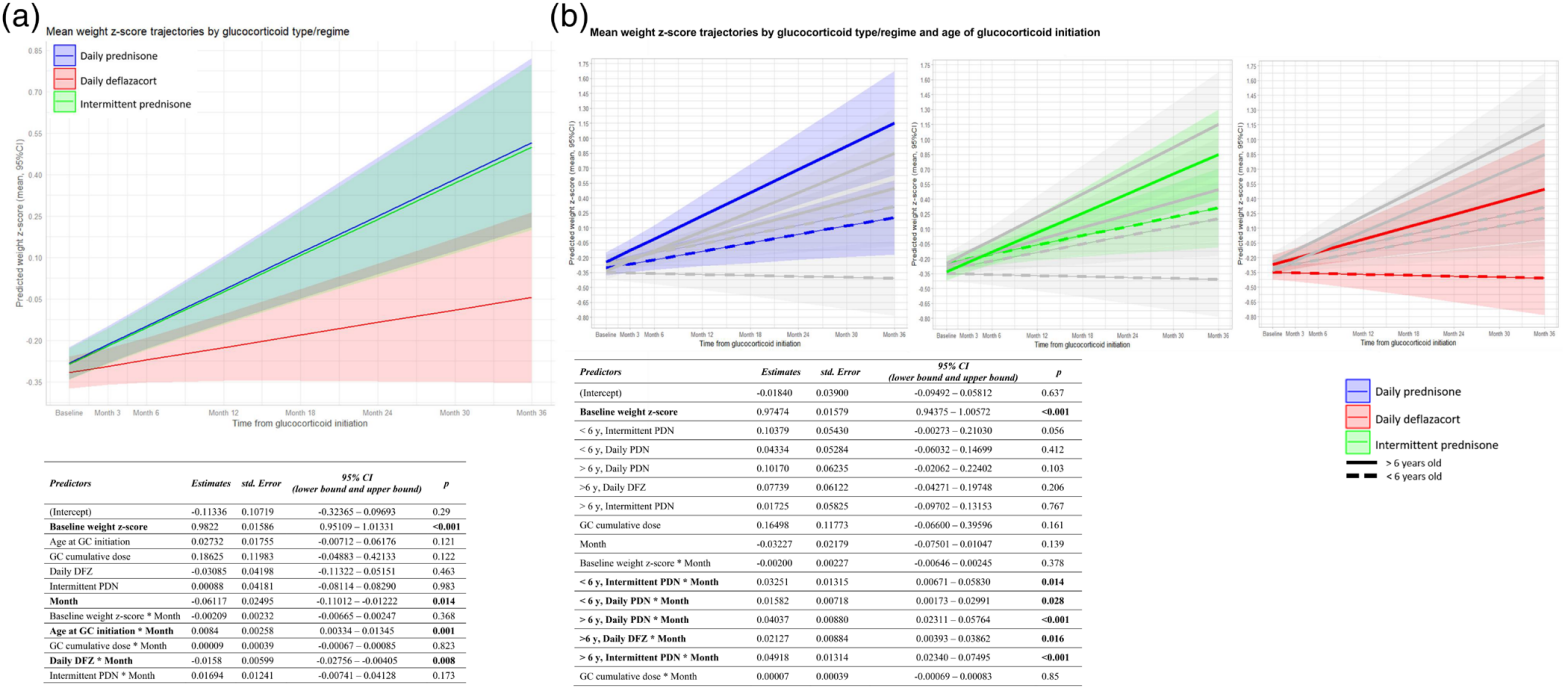
Weight z‐score trajectories. (a) Line graphs from the linear mixed‐effects model analysis showing weight z‐score trajectories over the 3‐year period of the FOR‐DMD study, categorized according to glucocorticoid regimen and type. The shading represents the confidence interval around the predicted trajectory, as estimated by the linear mixed‐effects model. The reference category consisted of boys on daily prednisone. (b) Line graphs from the linear mixed‐effects model analyses showing the weight z‐score trajectories over the 3‐year period of the FOR‐DMD study, categorized according to glucocorticoid regimen and type, and age at glucocorticoid initiation, either below or above 6 years old. The reference category consisted of boys younger than 6 years old on daily deflazacort. There were significant differences in the mean weight z‐scores over the 3‐year period between boys below or above 6 years old in the same glucocorticoid type and regime, except for boys on intermittent prednisone (mean [standard error] weight z‐score in the 3‐year period: less than 6 years daily PDN −0.180 [0.100] vs more than 6 years daily PDN 0.224 [0.131], mean difference − 0.403 [0.147], *p* = 0.033; less than 6 years daily DFZ −0.445 [0.101] vs more than 6 years daily DFZ −0.069 [0.134], mean difference − 0.376 [0.146], *p* = 0.053; less than 6 years intermittent PDN 0.115 [0.136] vs less than 6 years intermittent PDN 0.263 [0.140], mean difference 0.148 [0.138], *p* = 1). The variables in bold indicate those that achieved statistical significance in the model. The grey lines represent the trajectories of the other categories. Abbreviations: CI, confidence interval; DFZ, deflazacort; GC, glucocorticoid; PDN, prednisone/prednisolone.

**FIGURE 3:**
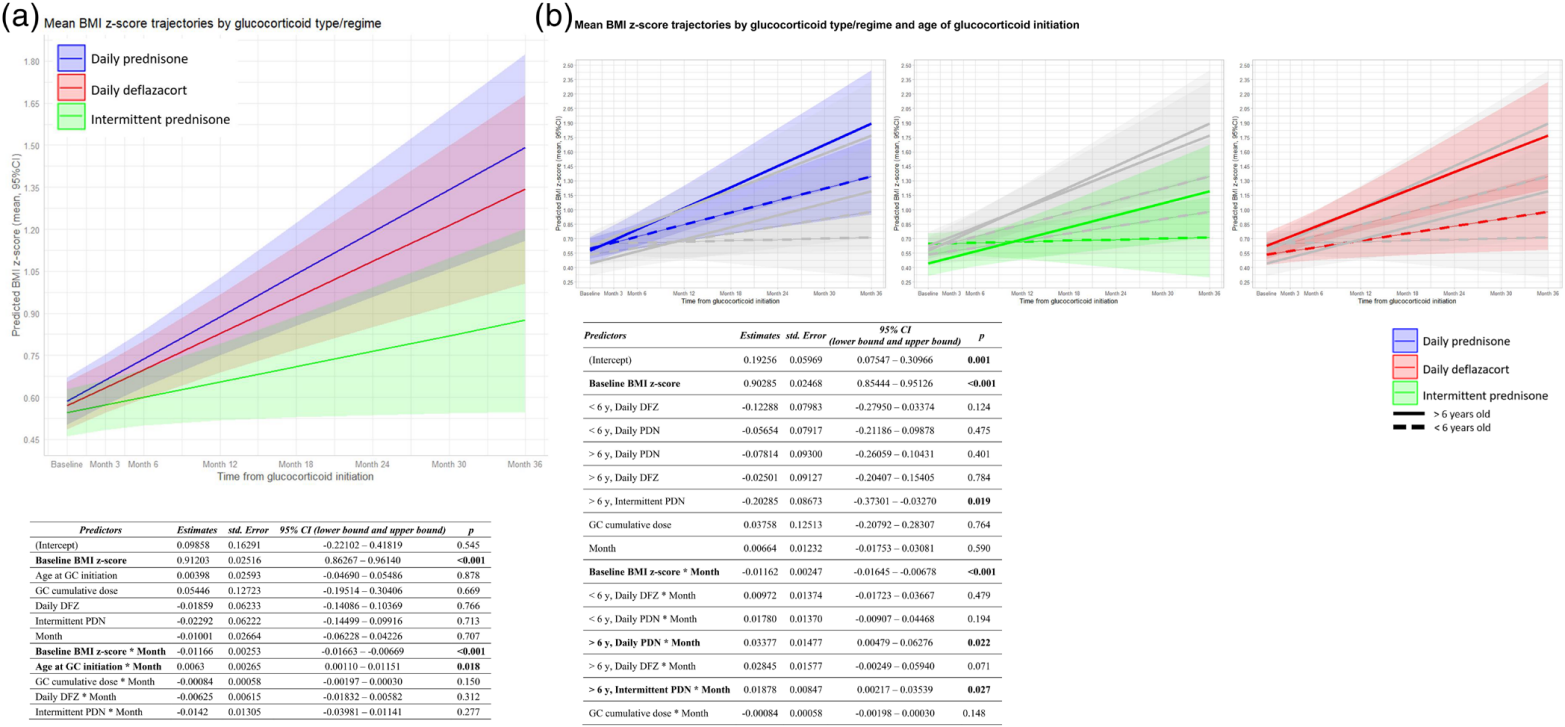
BMI z‐score trajectories. (a) Line graphs from the linear mixed model analysis showing the BMI z‐score trajectories over the 3‐year period of the FOR‐DMD study, categorized according to glucocorticoid regimen and type. The shading represents the confidence interval around the predicted trajectory, as estimated using the linear mixed model. The reference category consisted of boys on daily prednisone. (b) Line graphs from the linear mixed model analyses showing the BMI z‐score trajectories over the 3‐year period of the FOR‐DMD study, categorized according to glucocorticoid regimen and type, and age at glucocorticoid initiation, either younger or older than 6 years old. The reference category consisted of boys younger than 6 years old on intermittent prednisone. Despite boys younger than 6 years old showing lower numerical BMI z‐score values in all glucocorticoid type and regime categories, there were no significant differences in their mean BMI z‐scores (mean [standard error] weight z‐score in the 3‐year period: less than 6 years daily PDN 0.893 [0.110] vs more than 6 years daily PDN 1.094 [0.144], mean difference 0.201 [0.163], *p* = 1; less than 6 years daily DFZ 0.715 [0.111] vs more than 6 years daily DFZ 1.073 [0.147], mean difference 0.359 [0.162], *p* = 0.141; less than 6 years intermittent PDN 0.701 [0.147] vs more than 6 years intermittent PDN 0.761 [0.153], mean difference 0.058 [0.153], *p* = 1). The variables in bold indicate those that achieved statistical significance in the model. The grey lines represent the trajectories of the other categories. Abbreviations: BMI, body mass index; CI, confidence interval; DFZ, deflazacort; GC, glucocorticoid; PDN, prednisone/prednisolone.

The linear mixed‐effects model analyses showed that height, weight, and BMI z‐score followed a linear trajectory in the 3‐year period (Figures 1, 2, 3 and Appendix S2).

### Height z‐score trajectory

In 43.8% (85 of 194) of boys, the long dystrophin isoform (group 1, Dp427 absent/Dp140 and Dp71 present) was absent (Table 1). The dystrophin isoform did not influence the height z‐score trajectory, so it was excluded from further analysis (data not shown).

Baseline height z‐score and glucocorticoid type and regimen influenced height z‐score trajectories while age at glucocorticoid initiation and glucocorticoid cumulative dose did not, after accounting for glucocorticoid type and regime (linear mixed‐effects model, Figure 1).

Boys with a higher height z‐score at glucocorticoid initiation grew more slowly each month than boys with a lower height z‐score. For each additional unit in height z‐score at glucocorticoid initiation, the growth rate decreased by 0.006 z‐score units per month, after adjusting for other variables (*p* < 0.001; Figure 1a).

Even after adjusting for other variables in the model, the effect of glucocorticoid type and regime on the height z‐score trajectory was consistent with the previously reported height centile trajectories in the FOR‐DMD cohort (Figure 1a).^3^ Boys on daily deflazacort had greater growth stunting compared to boys on daily prednisone (estimated decrease in height growth rate of 0.016 z‐score units per month, *p <* 0.001). Conversely, boys on intermittent prednisone increased their height growth compared to boys on daily prednisone (estimated increment in height z‐score per month of 0.038, *p <* 0.001).

### Weight z‐score trajectory

The factors that influenced the weight z‐score trajectory included baseline weight z‐score, glucocorticoid regimen and type, and age at glucocorticoid initiation, whereas the glucocorticoid cumulative dose did not, after accounting for glucocorticoid regime (Figure 2).

Boys with DMD had a higher weight z‐score for their age at the time of glucocorticoid initiation (Figure 2a).

Older boys at glucocorticoid initiation gained more weight z‐score per month. For each additional year of age at the time of glucocorticoid initiation, there was an increase of 0.008 z‐score units per month, after adjusting for other variables (*p* = 0.001, Figure 2a). This finding was further supported by the model that categorized age at glucocorticoid initiation as either below or above the age of 6 years (Figure 2b).

Even after adjusting for other variables in the model, the effect of glucocorticoid type and regime on weight z‐score trajectories was consistent with the weight centile trajectory already reported in the FOR‐DMD cohort (Figure 2a).^3^ Boys on daily deflazacort had a lower increase in weight z‐score per month compared to boys on daily prednisone, with an estimated decrease of 0.015 z‐score units per month (*p* = 0.008). No differences were observed in the weight z‐score units gained per month between boys on daily and intermittent prednisone.

### BMI z‐score trajectory

Factors that influenced the BMI z‐score trajectory included baseline BMI z‐score and age at glucocorticoid initiation, whereas glucocorticoid type and regime and glucocorticoid cumulative dose did not (Figure 3).

Boys with a higher BMI z‐score at glucocorticoid initiation showed smaller monthly increases in BMI z‐score. For each 1‐unit increase in baseline BMI z‐score, there was an estimated decrease of 0.01 BMI z‐score units per month, after adjusting for other variables (*p <* 0.001) (Figure 3a).

**FIGURE 4:**
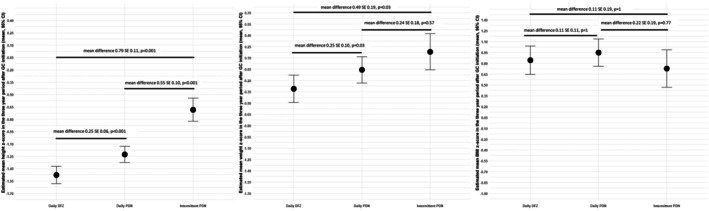
Mean anthropometric measures according to glucocorticoid regime and type up to 3 years of glucocorticoid initiation. Mean height, weight, and BMI z‐scores over the 3‐year period were compared across different glucocorticoid regimens and types adjusted according to the baseline z‐score, age at glucocorticoid initiation, and cumulative glucocorticoid dose. The means were calculated using the estimated marginal mean approach applied to the linear mixed‐effects models; Bonferroni corrections were applied for multiple comparisons. Abbreviations: BMI, body mass index; CI, confidence interval; DFZ, deflazacort; GC, glucocorticoid; PDN, prednisone/prednisolone.

Older boys at glucocorticoid initiation gained more BMI z‐score units per month. For each additional year of age at the time of glucocorticoid initiation, a boy's BMI z‐score increased 0.006 units per month (*p* = 0.018) (Figure 3a). The increase in BMI z‐score was related to the increase in the weight z‐score per month in older boys. As described previously, age at glucocorticoid initiation significantly influenced the weight z‐score trajectory (Figure 2) but did not affect the height z‐score trajectory (Figure 1). Although boys on daily deflazacort and intermittent prednisone had lower BMI than those on daily prednisone, the difference was not statistically significant after adjusting for other variables in the model (Figure 3a). Figure 4 shows the mean changes in height, weight, and BMI z‐score in the 3‐year period according to glucocorticoid regime and type after adjusting for age at glucocorticoid initiation, baseline z‐score, and glucocorticoid cumulative dose.

### Correlation between height, weight, and BMI z‐scores and COAs after 3 years of glucocorticoid treatment

Of the 194 boys, 59.79% (*n* = 116) remained on the study, fulfilling the percentage of target dose criteria specified for this analysis at 3 years of follow‐up. Fifteen boys were unable to rise from the floor and 10 lost ambulation. They were allocated a velocity of zero on the RFV and 10MWRV accordingly.

A Spearman's partial correlation analysis examined the relationship between changes in height, weight, or BMI z‐score and changes in COA over the 3‐year period, and between absolute z‐score values and COAs at year 3. Overall, all the correlations were weak.

The height z‐score change over the 3‐year period primarily influenced COA performance. A change in height z‐score showed a weak negative correlation with changes in all COAs (*p <* 0.05 in all cases) (Table 2). At year 3, the height z‐score correlated only with the 10MWRV (Spearman's rank correlation coefficient = −0.19, *p* = 0.043) but not with any other COAs (Table 2).

**TABLE 2:** Spearman's partial correlation between changes in z‐scores at the 3‐year period for height, weight, and BMI z‐scores and clinical outcome assessment after 3 years of glucocorticoid treatment.

Variable	Change in height z‐score, Spearman's rank correlation coefficient (95% CI)	Variable	Height z‐score, Spearman's rank correlation coefficient (95% CI)
Change in NSAA total score (*n* = 116)	−0.27 (−0.44 to −0.09)**	NSAA total score (*n* = 112)	−0.09 (−0.28 to 0.10)^NS^
Change in RFV (rise/second) (*n* = 116)	−0.30 (−0.46 to −0.12)**	RFV (rise per second) (*n* = 112)	−0.16 (−0.34 to 0.03)^NS^
Change in 10MWRV (10 m per second) (*n* = 116)	−0.21 (−0.38 to −0.02)*	10MWRV (10 m per second) (*n* = 110)	−0.19 (−0.37 to −0.0009)*
Change in 6MWT (m) (*n* = 114)	−0.22 (−0.39 to −0.03)*	6MWT (m) (*n* = 107)	−0.09 (−0.28 to 0.10)^NS^
	Change in weight z‐score, Spearman's rank correlation coefficient (95% CI)		Weight z‐score, Spearman's rank correlation coefficient (95% CI)
Change in NSAA total score (*n* = 116)	−0.26 (−0.43 to −0.08)**	NSAA total score (*n* = 116)	−0.21 (−0.38 to −0.02)*
Change in RFV (rise per second) (*n* = 116)	−0.38 (−0.53 to −0.21)***	RFV (rise per second) (*n* = 116)	−0.23 (−0.40 to −0.05)*
Change in 10MWRV (10 m per second) (*n* = 116)	−0.20 (−0.37 to −0.01)*	10MWRV (10 m per second) (*n* = 114)	−0.22 (−0.39 to −0.03)*
Change in 6MWT (m) (*n* = 114)	−0.26 (−0.43 to −0.08)**	6MWT (m) (*n* = 111)	−0.27 (−0.44 to −0.09)**
	Change in BMI z‐score, Spearman's rank correlation coefficient (95% CI)		BMI z‐score, Spearman's rank correlation coefficient (95% CI)
Change in NSAA total score (*n* = 116)	−0.11 (−0.29 to 0.08)^NS^	NSAA total score (*n* = 112)	−0.23 (−0.41 to −0.05)*
Change in RFV (rise per second) (*n* = 116)	−0.25 (−0.42 to −0.06)**	RFV (rise per second) (*n* = 112)	−0.20 (−0.38 to −0.01)*
Change in 10MWRV (10 m per second) (*n* = 116)	−0.10 (−0.29 to 0.09)^NS^	10MWRV (10 m per second) (*n* = 110)	−0.24 (−0.41 to −0.05)*
Change in 6MWT (m) (*n* = 114)	−0.16 (−0.34 to 0.03)^NS^	6MWT (m) (*n* = 107)	−0.33 (−0.50 to −0.15)***

* *p <* 0.05,

** *p <* 0.01,

*** *p <* 0.001;

NS, not significant (*p* > 0.05). Analysis adjusted according to age at glucocorticoid initiation, glucocorticoid cumulative dose, baseline COA performance, and glucocorticoid regime and type.

Abbreviations: 6MWT, 6‐minute walk test (m); 10MWRV, 10‐m walk/run velocity; BMI, body mass index; CI, confidence interval; COA, clinical outcome assessment; NSAA, NorthStar Ambulatory Assessment; RFV, rise from supine velocity.

Both the change in weight z‐score and the final weight z‐score affected COA performance. Change in weight z‐score showed a weak negative correlation with changes in all COAs (*p <* 0.05 in all cases) (Table 2). Similarly, at year 3, the weight z‐score correlated with performance in all COAs (*p <* 0.05 in all cases) (Table 2).

The final BMI z‐score value at year 3 was more influential on COAs than its change in the 3‐year period. The change in BMI z‐score only correlated with change in the RFV (Spearman's rank correlation coefficient = −0.25, *p <* 0.007) but did not correlate with any other COAs. Conversely, at year 3, the BMI z‐score had a weak negative correlation with performance in all COAs (*p <* 0.05 in all cases) (Table 2).

### Correlation between height, weight, and BMI z‐scores and COAs after 5 years of glucocorticoid treatment

At year 5 of follow‐up, 39 of the 116 boys included in the 3‐year cross‐sectional analysis were still under follow‐up. Seven of the 39 boys were excluded because their percentage of target dose of glucocorticoid decreased by more than 25% between baseline and year 5. Consequently, the Spearman's partial correlation 5‐year analysis included 32 boys for the NSAA, RFV, and 10MWRV. A 5‐year analysis for the 6‐minute walk test was not conducted because of low numbers.

The change in height and weight z‐scores during the 5‐year period influenced COA performance and the strength of the correlation increased to moderate.

The change in height z‐score had a moderate negative correlation with changes in the NSAA and 10MWRV (Spearman's rank correlation coefficient = −0.52, *p* = 0.007 and Spearman's rank correlation coefficient = −0.68, *p <* 0.001 respectively) (Table 3).

**TABLE 3:** Spearman's partial correlation between changes in z‐scores and changes in COA for height, weight, and BMI after 5 years of glucocorticoid treatment.

Variable	Change in height z‐score, Spearman's rank correlation coefficient (95% CI)	Variable	Height z‐score, Spearman's rank correlation coefficient (95% CI)
Change in NSAA total score (*n* = 26)	−0.52 (−0.76 to −0.15)**	NSAA total score (*n* = 25)	−0.003 (−0.41 to 0.40)^NS^
Change in RFV (rise per second) (*n* = 26)	−0.37 (−0.67 to 0.03)^NS^	RFV (rise per second) (*n* = 26)	0.05 (−0.36 to 0.44)^NS^
Change in 10MWRV (10 m per second) *n* = 26	−0.68 (−0.85 to −0.38)**	10MWRV (10 m per second) (*n* = 26)	−0.08 (−0.46 to 0.33)^NS^
	Change in weight z‐score, Spearman's rank correlation coefficient (95% CI)		Weight z‐score, Spearman's rank correlation coefficient (95% CI)
Change in NSAA total score (*n* = 27)	−0.54 (−0.77 to −0.19)**	NSAA total score (*n* = 25)	0.003 (−0.40 to 0.41)^NS^
Change in RFV (rise per second) (*n* = 27)	−0.35 (−0.65 to 0.04)^NS^	RFV (rise per second) (*n* = 27)	0.02 (−0.38 to 0.40)^NS^
Change in 10MWRV (10 m per second) (*n* = 27)	−0.43 (−0.70 to −0.04)**	10MWRV (10 m per second) (*n* = 27)	0.07 (−0.33 to 0.45)^NS^
	Change in BMI z‐score, Spearman's rank correlation coefficient (95% CI)		BMI z‐score, Spearman's rank correlation coefficient (95% CI)
Change in NSAA total score (*n* = 26)	−0.10 (−0.48 to 0.31)^NS^	NSAA total score (*n* = 25)	−0.05 (−0.45 to 0.36)^NS^
Change in RFV (rise per second) (*n* = 26)	−0.10 (−0.48 to 0.31)^NS^	RFV (rise per second) (*n* = 26)	−0.05 (−0.44 to 0.35)^NS^
Change in 10MWRV (10 m per second) (*n* = 26)	−0.04 (−0.43 to 0.36)^NS^	10MWRV (10 m per second) (*n* = 26)	0.21 (−0.20 to 0.56)^NS^

* *p* < 0.05,

** *p <* 0.01,

*** *p <* 0.001;

NS, not significant (*p* > 0.05).

Abbreviations: 10MWRV, 10‐m walk/run velocity; BMI, body mass index; CI, confidence interval; NSAA, NorthStar Ambulatory Assessment; RFV, rise from supine velocity.

Change in weight z‐score showed a moderately negative correlation with changes in the NSAA (Spearman's rank correlation coefficient = −0.54, *p* = 0.004) and a weak negative correlation with changes in the 10MWRV (Spearman's rank correlation coefficient = −0.43, *p <* 0.027) (Table 3).

A change in height or weight z‐score did not correlate with a change in RFV in the 5‐year period (Table 3).

Neither changes in BMI z‐score in the 5‐year period nor the final BMI z‐score at year 5 correlated with the COAs (Table 3).

A subsequent subanalysis including exclusively boys on daily glucocorticoids showed similar correlation results between z‐scores and COAs (Appendix S3). The correlation between height, weight, and BMI centiles with COA performance at years 3 and 5 also yielded similar results (Appendix S4).

## DISCUSSION

Concerns have been raised regarding reduced stature in boys with DMD after glucocorticoid initiation.^11^ Our study indicates that the height z‐score at glucocorticoid initiation, rather than age at treatment initiation, is a more significant predictor of the height trajectory. Early glucocorticoid treatment should not be delayed due to growth concerns. Instead, clinicians should consider how glucocorticoids might affect growth based on a boys's baseline height z‐score. Notably, boys with higher z‐scores at initiation may experience more stunting, probably because of nearing their prepubertal height limit.

Our study showed that boys with DMD initiated glucocorticoids with slightly above‐average weight, and age at initiation significantly influenced the weight z‐score trajectories. Older boys had greater weight gain over 3 years. These findings underscore the importance of proactive weight management and nutrition before glucocorticoid treatment, rather than focusing solely on weight control after glucocorticoid initiation, especially for older boys with DMD^25^ and those on prednisone, where higher weight gain is anticipated.^3^ These data also support considering daily deflazacort for boys with higher baseline weight z‐scores, given similar clinical outcomes.^3^ The association between older age and increased weight gain may be linked to reduced mobility, highlighting the need for tailored dietary interventions as boys age.

In our study, the cumulative glucocorticoid dose did not affect the height, weight, or BMI z‐score trajectories after adjusting for glucocorticoid regimen and type and other covariates. The NorthStar database found no link between glucocorticoid dose and BMI changes, but an association with height changes.^26^ Our study focused on boys in the FOR‐DMD trial, where doses were stable, reducing the impact of dose fluctuations. The potential effect of cumulative dose on height may be confounded by glucocorticoid regimen distribution, as only 34% were on the intermittent regimen. Our results suggest that glucocorticoid dose adjustments based on body weight do not affect height, weight, or BMI z‐scores, supporting the use of higher doses unless contraindicated by side effects.

We used z‐scores to describe anthropometric trajectories because z‐scores offer the advantage of quantifying extreme values.^27^ Centiles were used for the sensitivity analysis. The results from the centile‐based analysis were consistent with those from the z‐score analysis, confirming the robustness of the findings across different metrics.

There is interest in understanding how changes in, or absolute values of, anthropometric measures influence motor performance after glucocorticoid initiation. Previous studies examined the relationship between anthropometric measures and loss of ambulation.^13^, ^14^, ^15^, ^28^ To our knowledge, this is the first study to explore the correlation between height, weight, and BMI z‐scores and four commonly used COAs to monitor disease progression in DMD, over both short‐term (3 years) and long‐term (5 years) periods after glucocorticoid initiation. We showed that changes in height and weight over time, rather than absolute values, more significantly affected COA performance, especially over 5 years. These results suggest that as DMD progresses, managing weight is crucial for preserving motor function, particularly as height stabilizes while weight changes continue to influence performance.

Regarding the effect of the BMI z‐score, our findings align with an Australian study which found no correlation between BMI or BMI z‐score changes and COA performance.^29^ The lack of association may reflect the complex relationship between height and weight in DMD, and the varying effects of glucocorticoid types and regimens. In boys on intermittent prednisone, the effects of the height z‐score on the BMI z‐score may be balanced by an increase in the weight z‐score. No significant differences in BMI z‐score were found between boys on daily prednisone and boys on daily deflazacort, suggesting that the BMI z‐score in prednisone‐treated boys is driven by weight, while in deflazacort‐treated boys, it is influenced more by shorter stature.

This study supports clinical decisions for boys with DMD showing worsening in rising from the floor, the first milestone affected.^30^ A decline in this ability, even if other COAs remain stable and without confounding factors (e.g. lower‐leg contractures), may suggest that weight is a contributing factor. In such cases, weight management is crucial, maintaining the recommended glucocorticoid dose, and close monitoring is needed to determine if the decline is due to excess weight or actual muscle weakness. In this context, an assessment of the boys's overall motor performance should be considered, along with other COAs, such as the NSAA score or 6‐minute walk test (m), which could provide a more accurate understanding of their status before defining a decline in global motor function.

### Study limitations

Several factors not included in this analysis, such as pregnancy history, growth hormone levels, parental height and weight, and nutrition monitoring, could have influenced the anthropometric trajectories. A recent commentary by Birnkrant^31^ highlighted the presence of uncontrolled intrinsic (inherent to the patient) and extrinsic (related to external factors such as exposure to evolving standards of care) variables that can influence the outcome under evaluation. This underscores the importance of properly designing future natural history studies in DMD to minimize the impact of these uncontrolled variables. The FOR‐DMD study, while representing an appropriate setting for assessing anthropometric trajectories in boys with DMD and their correlation with COAs—because of closely monitored compliance with the glucocorticoid regimen, type and dose, protocolized management of adverse events, and measurements taken by trained clinical evaluators—was not without limitations. Additionally, the smaller number of boys followed at year 5 may have reduced the power of correlations at that time. Long‐term correlations between z‐scores and COAs need to be explored in larger cohorts. As switching glucocorticoid regimens and types was not allowed in the FOR‐DMD study, further analysis using real‐world data is needed to assess the impact of such changes on anthropometric measures and COA performance. Finally, despite the controversial evidence regarding a consistent and robust effect of genetic modifiers on motor performance in DMD,^32^ the impact of genetic modifiers in boys with DMD from the FOR‐DMD study has not yet been analysed, and the analysis in this manuscript was not adjusted for genetic modifiers.

## Conclusion

Our findings highlight factors affecting height, weight, and BMI z‐scores, and their impact on COA performance. This emphasizes the importance of monitoring weight changes and understanding how baseline traits influence growth and motor function.

## CONFLICT OF INTEREST STATEMENT

MS received a grant from UK Duchenne through Newcastle University.

MG is part of the Medical Research Council (UK) and TREAT‐NMD. MG participates in advisory boards for Pfizer, NS Pharma, Roche, Italfarmaco, and Santhera. She has research collaborations with Edgewise and Sarepta Therapeutics. She is or has been principal investigator for clinical trials with Roche, Italfarmaco, Edgewise, Genethon, Dyne, Santhera, ReveraGen, Summit, Pfizer, and PTC Therapeutics. She has received speaker honoraria from Italfarmaco, Dyne, Roche, and Novartis.

LMW declares consultancy work for Amgen, Ultragenyx, Kyowa Kirin, Angitia, Riche, Santhera, Catalyst, Biomarin, and Ipsen, which funds her institution. LMW also declares participation in clinical trials with Alexion, QED, Ultragenyx, Egdewise, ReveraGen, Ascendis, Roche, and Catalyst, which funds her institution.

RCG reports grants from the NIH, MDA, and Patient Project for Muscular Dystrophy Support and others from PTC Therapeutics and Sarepta Therapeutics, during the conduct of the study.

CLW has acted as a paid consultant to Roche and PTC Therapeutics.

UJD reports grants from the National Institutes of Health National Institute of Arthritis and Musculoskeletal and Skin Diseases and NINDS (US), the US Department of Defence, the Natural Sciences and Engineering Research Council of Canada, and the Foundation to Eradicate Duchenne.

UJD declares consultancy for ReveraGen Biopharma, Lupin Neurosciences, and iuvo Bioscience, and declares participation in clinical trials with ReveraGen Biopharma.

AM has participated in SAB meetings for Summit, PTC Therapeutics, and Biogen and performs consultancy work (training physiotherapists for a trial in DMD) for Roche, Pfizer, PTC Therapeutics, Summit, Sarepta Therapeutics, Santhera, Italfarmaco, Amicus, Biogen, and Avexis.

SCW declares consultancy work for Novartis, Santhera, and Roche, and has received speaker's honoraria from Sandoz, Roche, Novo Nordisk, and Nutricia. The other authors declare no disclosures related to this project.

## Supporting information


**Appendix S1:** Vitamin D, calcium, and phosphate serum levels through the 3‐year study period

**Appendix S2:** Estimated mean height, weight and BMI z‐score trajectories and predicted individual trajectories by glucocorticoid regimen and type in the three‐year period.

**Appendix S3:** Partial correlation analysis in boys exclusively on daily glucocorticoid regimens

**Appendix S4:** Partial correlation between anthropometric measurement (percentile for age and sex) and clinical outcome assessments.

## Data Availability

The first author and corresponding author have full access to the data used in the analyses for this manuscript. The first author takes full responsibility for the data, the analyses, interpretation, and the conduct of the research. The first and corresponding author have the right to publish any and all data. De‐identified participant data will be archived on the NINDS website for archived clinical research datasets (URL number not available yet) from 08‐31‐2023.
