# Supplementary material for: Height, weight, and body mass index trajectories and their correlation with functional outcome assessments in boys with Duchenne muscular dystrophy
Source: Dev Med Child Neurol. 2025 Aug 31;68(3):429–40. doi: 10.1111/dmcn.16437 (PMC12875185; doi:10.1111/dmcn.16437)
Supplement: Supplementary file 1 — Appendix S1: Vitamin D, calcium, and phosphate serum levels through the 3‐year study period [file DMCN-68-429-s004.docx]

**Appendix 1**

**Appendix 1.a Vitamin D, calcium, and phosphate serum levels through the 3-year study period.**

| **Timepoint** | **N** | **Vitamin D [nmol/L]**  **(mean, SD, median, minimum – maximum)** | **N** | **Calcium [mg/dL]**  **(mean, SD, median, minimum – maximum)** | **N** | **Phosphate [mg/dL]**  **(mean, SD, median, minimum – maximum)** | **N** | **Alkaline phosphatase [IU/L]**  **(mean, SD, median, minimum – maximum)** |
| --- | --- | --- | --- | --- | --- | --- | --- | --- |
| Baseline | 190 | 47.36 + 20.75  45.13 (10.00 – 164.75) | 192 | 9.45 + 0.96  9.61 (4.00 - 11.00) | 169 | 4.97 + 0.44  5.00 (3.84 - 06.12) | 192 | 152.34 + 67.33  132.00 (74.00 – 460.00) |
| Month 12 | 161 | 59.16 + 25.00  54.70 (20.00 – 131.25) | 163 | 9.65 + 0.51  9.70 (4.81 - 10.90) | 136 | 4.77 + 0.61  4.71 (3.00 - 07.43) | 164 | 122.71 + 57.69  107.00 (53.00 – 385.00) |
| Month 24 | 124 | 59.84 + 26.24  54.13 (17.20 – 177.25) | 132 | 9.61 + 0.50  9.62 (5.50 - 10.60) | 111 | 4.78 + 0.61  4.80 (2.51 - 07.06) | 132 | 124.54 + 99.87  104.00 (44.00 – 1080.00) |
| Month 36 | 96 | 64.36 + 24.50  60.45 (18.90 – 147.50) | 103 | 9.63 + 0.64  9.66, (4.69 - 12.30) | 89 | 4.69 + 0.53  4.65 (3.34 - 05.70) | 104 | 108.66 + 45.86  100.50 (34.00 – 290.00) |

**Appendix 1. Vitamin D serum levels categorized by groups and vitamin D replacement throughout the 3-year study period.**

**
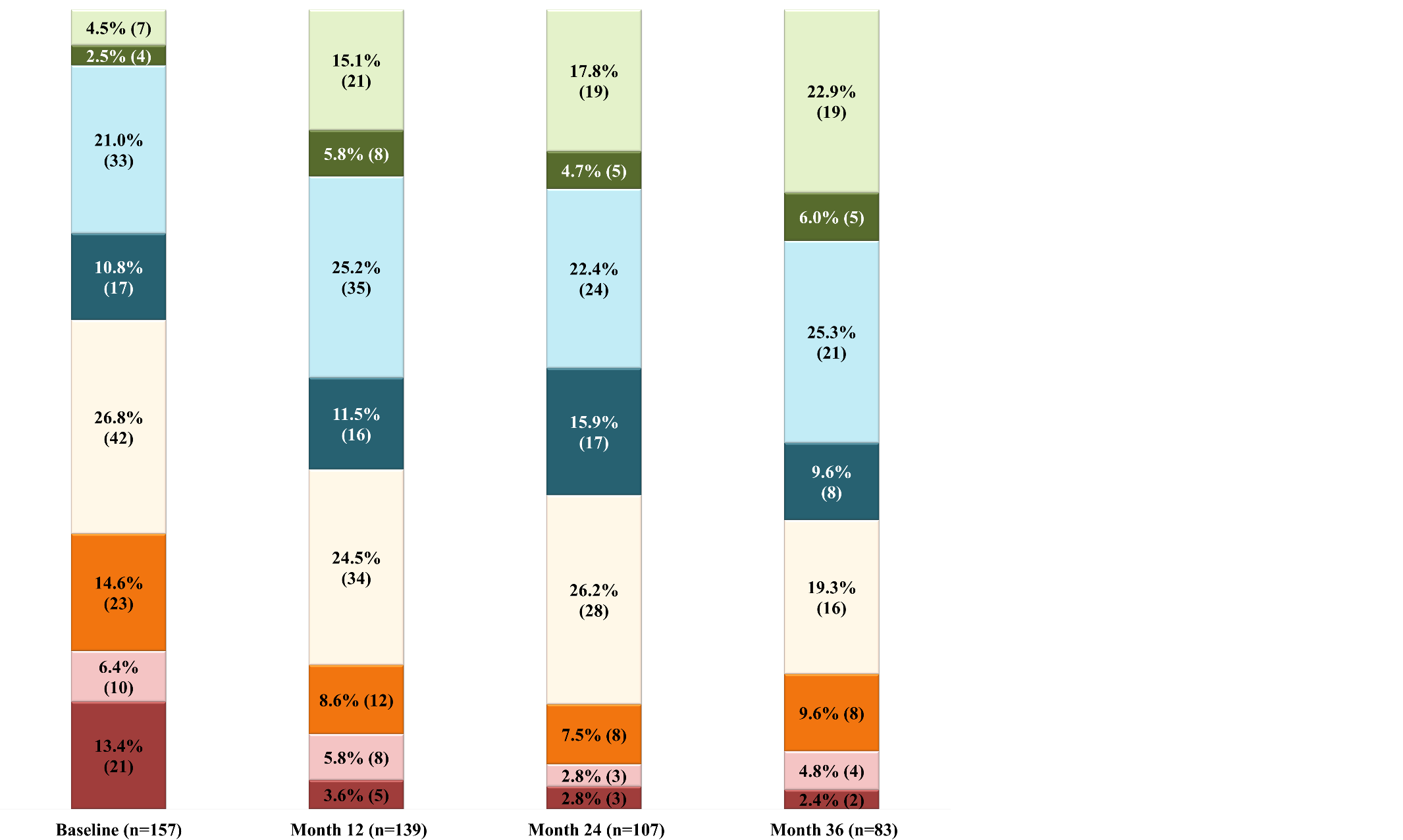
**


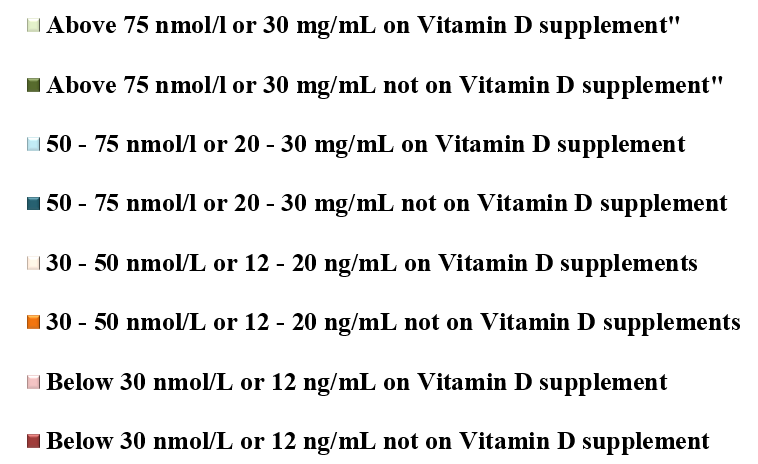


Serum concentrations of 25(OH)D are reported in both nanomoles per Liter (nmol/L) and nanograms per millilitre (ng/mL). One nmol/L = 0.4 ng/mL, and 1 ng/mL = 2.5 nmol/L. Numbers in brackets represents number of boys.

The solid blue line indicates a serum Vitamin D level <30 nmol/L or < 12 ng/mL generally associated with vitamin D deficiency, which can lead to rickets in infants and children and osteomalacia in adults.

The dashed line indicates a serum Vitamin D level ≥50 nmol/L or ≥20 ng/mL generally considered adequate for bone and overall health in healthy individuals (lowest acceptable Vitamin D level).

The solid line indicates a serum Vitamin D level ≥75 nmol/L or ≥30 ng/mL which is the ideal Vitamin D target.

Among all the boys who had a Vitamin D level <30 nmol/L or < 12 ng/mL, in only four cases the calcium level was below the normal limit of normal for children (child normal range: 8.8-10.8 mg/dL or 2.2-2.7 mmol/L) and the four had normal values of phosphate (Child normal range: 4.5-6.5 mg/dL or 1.45-2.1 mmol/L).

Reference: Health NI of O of dietary supplements. Vitamin D - Fact Sheet for Health Professionals. Published 2024. Accessed August 22, 2024. Available on: https://ods.od.nih.gov/factsheets/VitaminD-HealthProfessional/#:~:text=One nmol%2FL %3D 0.4 ng,mL %3D 2.5 nmol%2FL.
