# Supplementary material for: Height, weight, and body mass index trajectories and their correlation with functional outcome assessments in boys with Duchenne muscular dystrophy
Source: Dev Med Child Neurol. 2025 Aug 31;68(3):429–40. doi: 10.1111/dmcn.16437 (PMC12875185; doi:10.1111/dmcn.16437)
Supplement: Supplementary file 2 — Appendix S2: Estimated mean height, weight and BMI z‐score trajectories and predicted individual trajectories by glucocorticoid regimen and type in the three‐year period. [file DMCN-68-429-s003.docx]

**Appendix S2**

**Figure 1. Estimated mean height z-score trajectories and predicted individual trajectories by glucocorticoid regimen and type in the three-year period.**


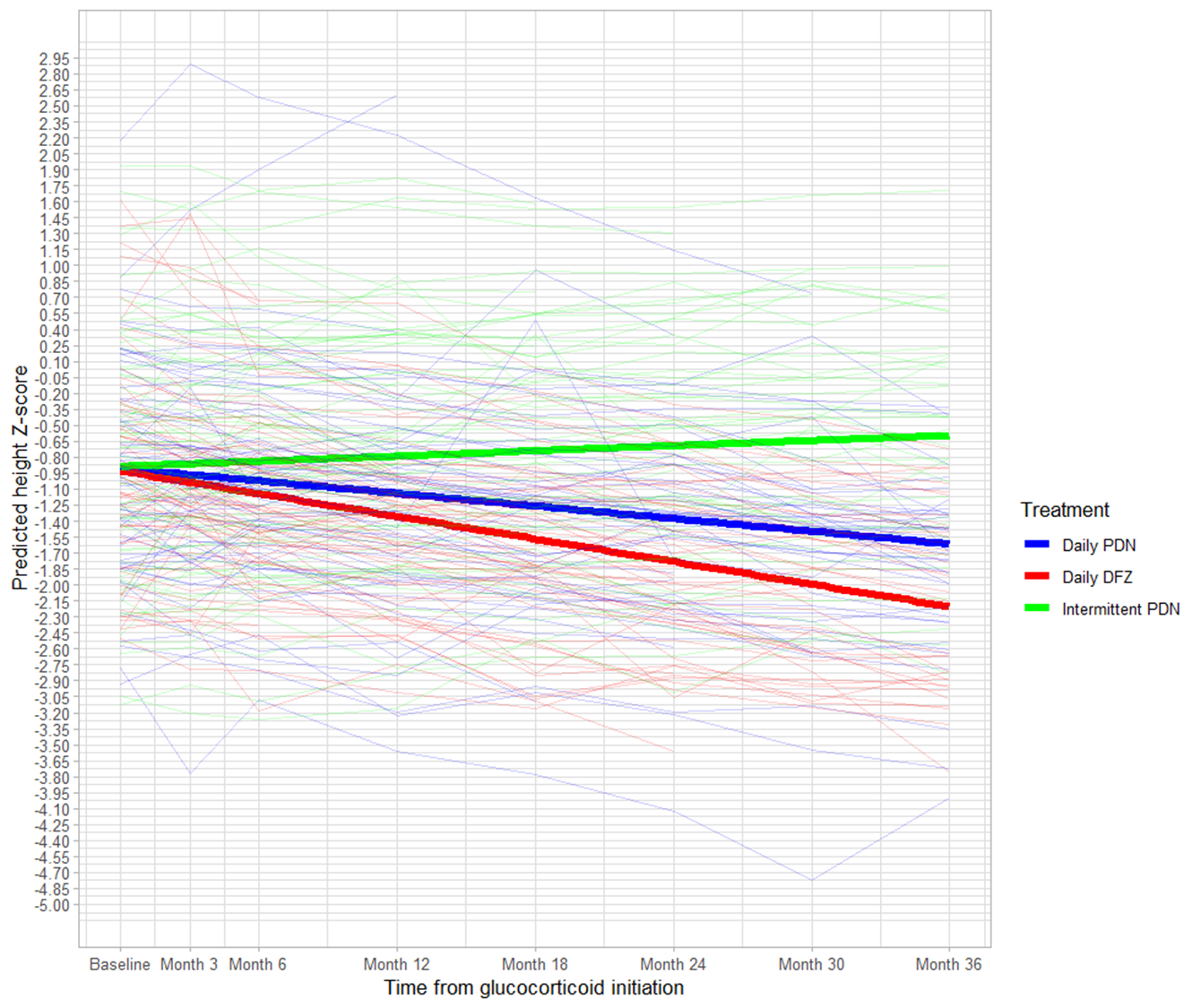


Line graphs from the linear mixed model analyses illustrate the estimated mean height z-score trajectories over the three-year period of the FOR-DMD study, categorized by glucocorticoid type and regimen. Thinner lines represent the model-predicted trajectories for individual participants. The reference group comprises boys receiving a daily prednisone regimen. DFZ: deflazacort. PDN: prednisone/prednisolone.

**Figure 2. Estimated mean weight z-score trajectories and predicted individual trajectories by glucocorticoid regimen and type in the three-year period.**


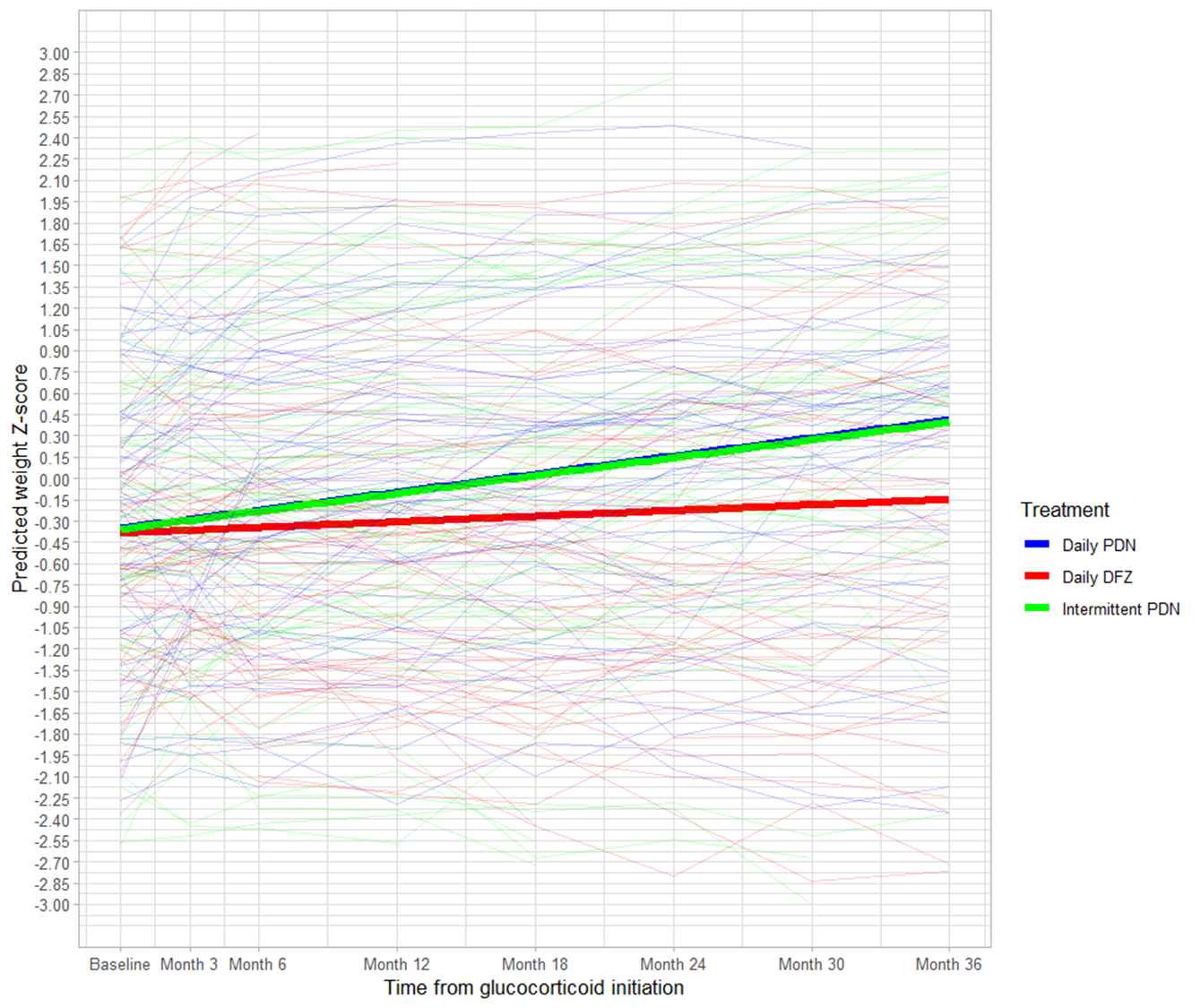


Line graphs from the linear mixed model analyses illustrate the estimated mean weight z-score trajectories over the three-year period of the FOR-DMD study, categorized by glucocorticoid type and regimen. Thinner lines represent the model-predicted trajectories for individual participants. The reference group comprises boys receiving a daily prednisone regimen. DFZ: deflazacort. PDN: prednisone/prednisolone.

**Figure 3. Estimated mean Body Mass Index z-score trajectories and predicted individual trajectories by glucocorticoid regimen and type in the three-year period.**


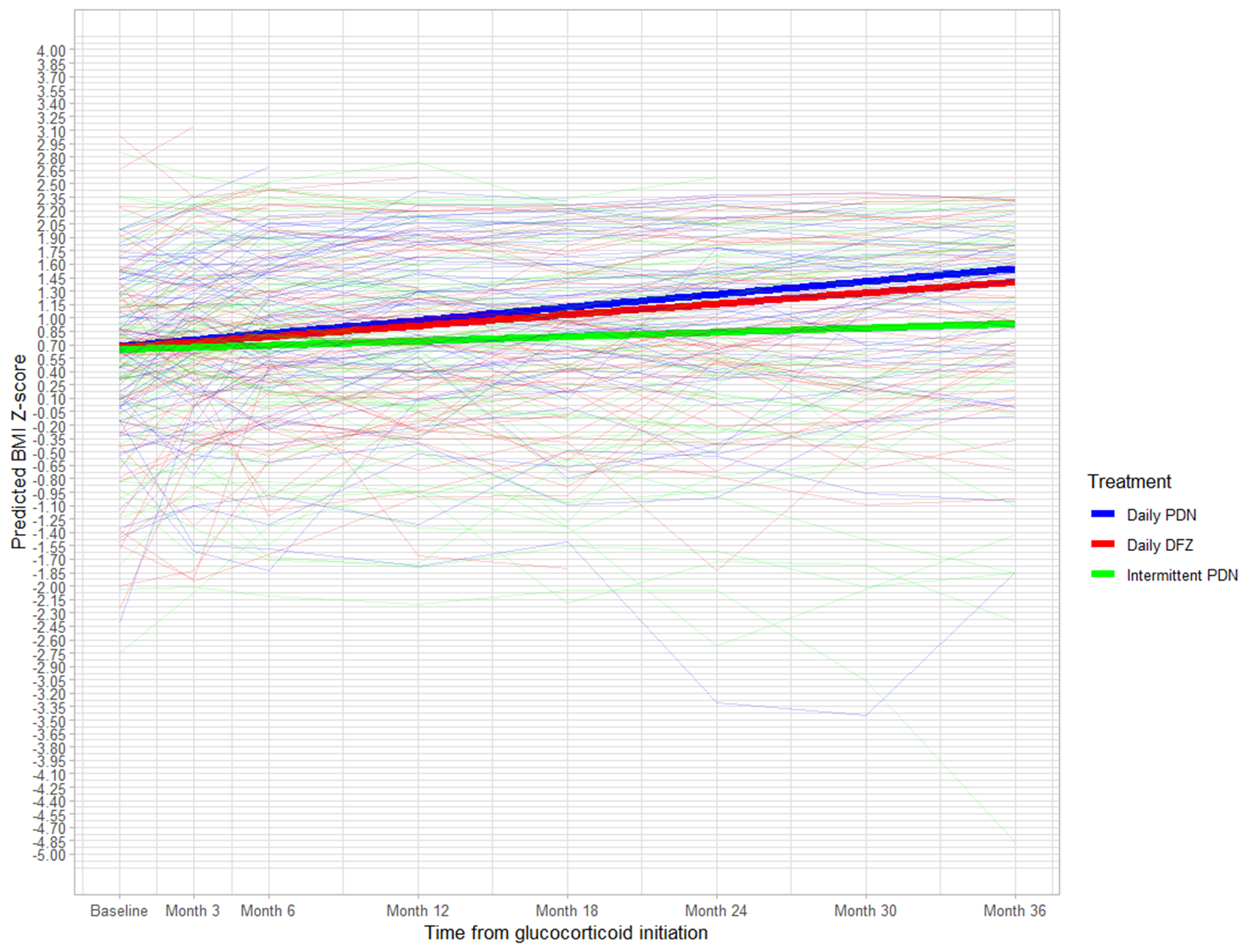


Line graphs from the linear mixed model analyses illustrate the estimated mean BMI z-score trajectories over the three-year period of the FOR-DMD study, categorized by glucocorticoid type and regimen. Thinner lines represent the model-predicted trajectories for individual participants. The reference group comprises boys receiving a daily prednisone regimen. DFZ: deflazacort. PDN: prednisone/prednisolone. BMI: body mass index.
