# Supplementary material for: Height, weight, and body mass index trajectories and their correlation with functional outcome assessments in boys with Duchenne muscular dystrophy
Source: Dev Med Child Neurol. 2025 Aug 31;68(3):429–40. doi: 10.1111/dmcn.16437 (PMC12875185; doi:10.1111/dmcn.16437)
Supplement: Supplementary file 3 — Appendix S3: Partial correlation analysis in boys exclusively on daily glucocorticoid regimens [file DMCN-68-429-s001.docx]

|  | **Partial correlation between changes in anthropometric measurement and changes in COAs in the three-year period**  **boys on daily regimes** |  | **Partial correlation between anthropometric measurement and COAs at year three boys on daily regimes** |
| --- | --- | --- | --- |
| **Variables correlated** | **Change in height z-score**  Spearman ρ [95% CI] |  | **Height z-score**  Spearman ρ [95% CI] |
| Change in NSAA total score  n=79 | **-0.25 [-0.45, -0.03] ^*^** | NSAA total score  n=76 | 0.21 [-0.25, 0.60] ^ns^ |
| Change in RFV (rise/sec)  n=79 | **-0.24 [-0.44, -0.01] ^*^** | RFV (rise/sec)  n=76 | **-0.25 [-0.46, -0.02] *** |
| Change in 10MWRV (10m/sec)  n=79 | **-0.25 [-0.45, -0.02] ^*^** | 10MWRV (10m/sec) n=76 | **-0.23 [-0.44, −0.0002] *** |
| Change in 6MWT (metres)  n=79 | **-0.29 [-0.49, -0.06] ^*^** | 6MWT (metres)  n=76 | -0.14 [-0.36, 0.10] ^ns^ |
|  |  |  |  |
|  | **Change in weight z-score**  Spearman ρ [95% CI] |  | **Weight z-score**  Spearman ρ [95% CI] |
| Change in NSAA total score  n=79 | **-0.26 [-0.46, -0.04] ^*^** | NSAA total score  n=76 | -0.12 [-0.53, 0.34] ^ns^ |
| Change in RFV (rise/sec)  n=79 | **-0.41 [-0.58, -0.20] ^***^** | RFV (rise/sec)  n=76 | **-0.27 [-0.47, -0.05] *** |
| Change in 10MWRV (10m/sec)  n=79 | **-0.23 [-0.44, -0.01] ^*^** | 10MWRV (10m/sec) n=76 | **-0.24 [-0.44, -0.01] *** |
| Change in 6MWT (metres)  n=79 | **-0.35 [-0.53, -0.13] ^**^** | 6MWT (metres)  n=76 | **-0.24 [-0.45, -0.01] *** |
|  |  |  |  |
|  | **Change in BMI z-score**  Spearman ρ [95% CI] |  | **BMI z-score**  Spearman ρ [95% CI] |
| Change in NSAA total score  n=79 | -0.06 [-0.29, 0.17] ^ns^ | NSAA total score  n=76 | -0.22 [-0.60, 0.25] ^ns^ |
| Change in RFV (rise/sec)  n=79 | **-0.3 [-0.49, -0.08] ^**^** | RFV (rise/sec)  n=76 | -0.22 [-0.43, 0.01] |
| Change in 10MWRV (10m/sec)  n=79 | -0.09 [-0.31, 0.14] ^ns^ | 10MWRV (10m/sec) n=76 | **-0.23 [-0.44, 0.00] *** |
| Change in 6MWT (metres)  n=79 | -0.15 [-0.37, 0.08] ^ns^ | 6MWT (metres)  n=76 | **-0.26 [-0.47, -0.02] *** |

|  | **Partial correlation between changes in anthropometric measurement and changes in COAs in the five-year period boys on daily regimes** |  | **Partial correlation between anthropometric measurement and COAs at year five boys on daily regimes** |
| --- | --- | --- | --- |
| **Variables correlated** | **Change in height z-score**  Spearman ρ [95% CI] |  | **Height z-score**  Spearman ρ [95% CI] |
| Change in NSAA total score  n=21 | -0.39 [-0.71, 0.06] ^ns^ | NSAA total score  n=21 | 0.21 [-0.25, 0.60] ^ns^ |
| Change in RFV (rise/sec)  n=21 | **-0.48 [-0.7584, -0.041] *** | RFV (rise/sec)  n=21 | 0.16 [-0.30, 0.56] ^ns^ |
| Change in 10MWRV (10m/sec)  n=21 | **-0.66 [-0.85, -0.31] **** | 10MWRV (10m/sec)  n=21 | 0.21 [-0.2524, 0.60] ^ns^ |
|  |  |  |  |
|  | **Change in weight z-score**  Spearman ρ [95% CI] |  | **Weight z-score**  Spearman ρ [95% CI] |
| Change in NSAA total score  n=21 | **-0.51[-0.78, -0.09] *** | NSAA total score  n=21 | -0.12 [-0.53, 0.34] ^ns^ |
| Change in RFV (rise/sec)  n=21 | -0.4 [-0.72, 0.05] ^ns^ | RFV (rise/sec)  n=21 | -0.14 [-0.55, 0.32] ^ns^ |
| Change in 10MWRV (10m/sec)  n=21 | **-0.6 [-0.83, -0.22] **** | 10MWRV (10m/sec)  n=21 | 0.00779 [-0.44, 0.45] ^ns^ |
|  |  |  |  |
|  | **Change in BMI z-score**  Spearman ρ [95% CI] |  | **BMI z-score**  Spearman ρ [95% CI] |
| Change in NSAA total score  n=21 | 0.32 [-0.67, 0.14] ^ns^ | NSAA total score  n=21 | -0.22 [-0.60, 0.25] ^ns^ |
| Change in RFV (rise/sec)  n=21 | -0.08 [-0.51, 0.38] ^ns^ | RFV (rise/sec)  n=21 | -0.10 [-0.52, 0.36] ^ns^ |
| Change in 10MWRV (10m/sec)  n=21 | -0.37 [-0.70, 0.09] ^ns^ | 10MWRV (10m/sec)  n=21 | -0.06 [-0.49, 0.40] ^ns^ |
| ^***^ p<0.001; ^**^ p<0.01; ^*^ p<0.05; ^ns^ not significant (p>0.05).  RFV: rise from supine velocity, rise/s. 10MWRV: 10-meter walk/run velocity, 10m/s. NSAA: North Star Ambulatory Assessment total score. 6MWT: six-minute walk test, meters. BMI: body mass index. | | | |
