# Supplementary material for: Height, weight, and body mass index trajectories and their correlation with functional outcome assessments in boys with Duchenne muscular dystrophy
Source: Dev Med Child Neurol. 2025 Aug 31;68(3):429–40. doi: 10.1111/dmcn.16437 (PMC12875185; doi:10.1111/dmcn.16437)
Supplement: Supplementary file 4 — Appendix S4: Partial correlation between anthropometric measurement (percentile for age and sex) and clinical outcome assessments. [file DMCN-68-429-s002.docx]

| **Partial correlation between anthropometric measurement (percentile for age and sex)**  **and clinical outcome assessments** | | |
| --- | --- | --- |
| **Variables correlated** | **3 years of follow up** | **5 years of follow up** |
|  | **Height percentile** Spearman ρ [95% CI] | |
| NSAA total score | -0.03 [-0.22, 0.16] ^ns^ | -0.003 [-0.41, 0.40] ^ns^ |
| RFV (rise/sec) | -0.07 [-0.26, 0.12] ^ns^ | 0.05 [-0.36, 0.44] ^ns^ |
| 10MWRV (10m/sec) | -0.13 [-0.31, 0.07] ^ns^ | -0.08 [-0.46, 0.33] ^ns^ |
| 6MWT (metres) | -0.05 [-0.24, 0.15] ^ns^ | NA |
|  |  |  |
|  | **Weight percentile** Spearman ρ [95% CI] | |
| NSAA total score | **-0.21 [-0.38, -0.03] ^*^** | 0.03 [-0.38, 0.43] ^ns^ |
| RFV (rise/sec) | **-0.24 [-0.41, -0.05] ^*^** | -0.03 [-0.41, 0.37] ^ns^ |
| 10MWRV (10m/sec) | **-0.22 [-0.39, -0.03] ^*^** | 0.07 [-0.33, 0.44] ^ns^ |
| 6MWT (metres) | **-0.26 [-0.43, -0.07] ^**^** | NA |
|  |  |  |
|  | **BMI percentile** Spearman ρ [95% CI] | |
| NSAA total score | **-0.23 [-0.40, -0.04] ^*^** | -0.02 [-0.42, 0.39] ^ns^ |
| RFV (rise/sec) | -0.17 [-0.35, 0.02] ^ns^ | -0.01 [-0.41, 0.39] ^ns^ |
| 10MWRV (10m/sec) | **-0.22 [-0.40, -0.03] ^*^** | 0.2 [-0.21, 0.56] ^ns^ |
| 6MWT (metres) | **-0.29 [0.46, -0.10] ^**^** | NA |
| ^***^ p<0.001; ^**^ p<0.01; ^*^ p<0.05; ^ns^ not significant, p>0.05. NA: not applicable. ρ spearman rho.  RFV: rise from supine velocity, rise/s. 10MWRV: 10-meter walk/run velocity, 10m/s. NSAA: North Star Ambulatory Assessment total score. 6MWT: six-minute walk test, meters. BMI: body mass index. | | |
